# MiR-942-3p as a Potential Prognostic Marker of Gastric Cancer Associated with AR and MAPK/ERK Signaling Pathway

**DOI:** 10.3390/cimb44090263

**Published:** 2022-08-24

**Authors:** Wenjia Liu, Nanjiao Ying, Xin Rao, Xiaodong Chen

**Affiliations:** 1 School of Electronics and Information Engineering, Hangzhou Dianzi University, Hangzhou 310018, China; 2 School of Automation, Hangzhou Dianzi University, Hangzhou 310018, China; 3 School of Electronic Engineering and Computer Science, Queen Mary University of London, London E1 4NS, UK

**Keywords:** gastric cancer, prognosis, biomarker, hsa-miR-942-3p, AR, MAPK/ERK signaling pathway

## Abstract

Gastric cancer is a common tumor with high morbidity and mortality. MicroRNA (miRNA) can regulate gene expression at the translation level and various tumorigenesis processes, playing an important role in tumor occurrence and prognosis. This study aims to screen miRNA associated with gastric cancer prognosis as biomarkers and explore the regulatory genes and related signaling pathways. In this work, R language was used for the standardization and differential analysis of miRNA and mRNA expression profiles. Samples were randomly divided into a testing group and a training group. Subsequently, we built the five miRNAs (has-miR-9-3p, has-miR-135b-3p, has-miR-143-5p, has-miR-942-3p, has-miR-196-3p) prognostic modules, verified and evaluated their prediction ability by the Cox regression analysis. They can be used as an independent factor in the prognosis of gastric cancer. By predicting and analyzing potential biological functions of the miRNA target genes, this study found that the AR gene was not only a hub gene in the PPI network, but also associated with excessive survival of patients. In conclusion, this study demonstrated that hsa-miR-942-3p could be a potential prognostic marker of gastric cancer associated with the AR and MAPK/ERK signaling pathways. The results of this study provide insights into the occurrence and development of gastric cancer.

## 1. Introduction

Gastric cancer is one of the most common tumors and its overall survival rate is only about 10% [1]. Some treatments are developing rapidly, including surgery, radiotherapy, chemotherapy, and targeted therapy. However, the recurrence rate and poor prognosis remain a troubling issue. At present, some biomarkers related to the occurrence and prognosis of gastric cancer have been found [2] but their reliability has not been completely verified. Therefore, it is essential to screen new biomarkers or therapeutic targets for the prognosis of gastric cancer patients.

MicroRNA (miRNA) is a non-coding molecule, which can regulate gene expression at the translation level. Some studies have shown that miRNAs regulate various tumorigenesis processes (cell proliferation, cell differentiation, and cell apoptosis) by combining tumor suppressor genes or oncogenes. Yang L et al. found that miR-9-3p was a down-regulated gene of glioma cells. Its low expression resulted in increased levels of Herpud1 that could protect glioma cells from apoptosis [3]. Chen Z et al. showed that miR-143-5p could promote cadmium-induced apoptosis of LLC-PK1 cells by acting on the target gene AKT3 and inhibiting the Akt/Bad signaling pathway [4]. Ma R et al. verified that up-regulated miR-196b could induce a proliferative phenotype, leading to a poor prognosis in glioblastoma patients [5]. Chen M et al. showed that miR-135b could play the role of oncogenes by regulating the PI3K/Akt, HIF-1/FIH, Hippo, p53 signaling pathways, promote tumor cell proliferation, migration, invasion, promote tumor angiogenesis, affect the prognosis of tumor patients, and reduce the total survival and survival time. Moreover, the expression of miR-135b in serum can be used as a biomarker for the diagnosis of a tumor [6]. In addition, miRNA also plays a great role in the treatment of gastric cancer [7]. Lin A et al. concluded that miRNA-449b was associated with the occurrence of gastric cancer and lymph node metastasis [8]. Ma X et al. found that the expression level of miRNA-375 in gastric cancer was related to the degree of tumor differentiation, which could be considered a clinical monitoring target [9]. Han W and Su X found that miRNA-30c showed low expression in gastric cancer tissues and was involved in the occurrence and development of gastric cancer by changing cell proliferation, apoptosis, and cell cycle [10]. With this in mind, the studies of miRNA in gastric cancer still need to be pushed forward and further investigated.

In this study, we constructed, validated, and evaluated five miRNAs and the results showed that they could be used as independent prognostic factors in gastric cancer. More importantly, we detected the target gene AR of hsa-miR-942-3p which was the core target gene and closely related to the prognosis and survival of gastric cancer patients. In short, hsa-miR-942-3p may be a potential prognostic marker of gastric cancer related to the AR and mitogen-activated protein kinase (MAPK)/extracellular signal-regulated kinase (ERK) signaling pathways.

## 2. Materials and Methods

### 2.1. Data Downloading and Processing

The miRNA and mRNA profiles data were gained from The Cancer Genome Atlas (TCGA) database (https://www.cancer.gov, accessed on 20 August 2022 (Table 1). The miRNA expression profiles included 45 normal and 446 tumor samples, and the mRNA expression profiles included 32 normal and 375 tumor samples. Clinical information (443) for all gastric cancer samples was also downloaded (Table 2).

### 2.2. Detection of Differentially Expressed miRNAs and mRNA Combined with Clinical Information

Standardization and differential analysis of expression profiles were performed using R language (*p* < 0.05 and |logFC| > 1.0) [11]. Thereafter, clinical information on patients was combined with the disposed of miRNAs and mRNAs.

### 2.3. Construction of Sample Grouping and Prognostic Module

Samples were divided into training group and testing group randomly by R language package. Univariate Cox regression analysis was used to detect the miRNAs with *p* < 0.05 in the training group. Multivariate Cox regression was used to build the miRNA module prognostic biomarkers with different overall survival [12]. Then, we established the risk score of a prognostic miRNA signature and detected the Proportional Hazards Assumption of the Cox module. The module was used to assess the survival prognosis of patients in three groups by the Kaplan–Meier curve. Log-rank tests were classified into a high-risk and low-risk group according to the risk score of the median value grouping. R language (“survivalROC” package) was used to evaluate miRNA predictive power by receiver operating characteristic (ROC) curve [13].

### 2.4. Independent Prognostic Ability of miRNA

The univariate Cox regression was analyzed to test the relationship between the prognostic miRNA and the overall survival of patients in the training group. Clinical factors were also analyzed by multivariate Cox regression to serve as independent prognostic elements.

### 2.5. miRNA Target Genes Prediction and Functions Analysis

The miRNA information was downloaded from three prediction databases (targetScan, miRTarBase, and miRDB). The target genes of miRNA were obtained and crosschecked in at least two databases. Using the Cytoscape and Venn software to draw the relation between miRNAs and the target genes. Differentially expressed genes (DEGs) and target genes were taken at the intersection to test whether these target genes were involved in the progression of gastric cancer. Kyoto Encyclopedia of Genes and Genomes (KEGG) enrichment pathway and Gene Ontology (GO) analysis displayed the potential function of all the intersection genes through R language (“org.Hs.eg.db” package and “clusterProfiler” package) [14].

### 2.6. Screening Core Target Genes and Survival Analysis

The protein–protein interaction (PPI) network between the target genes was obtained from STRING websites [15] while the medium confidence is 0.400. Then, the top ten hub genes were detected through Cytoscape plug-in CytoHubba. In addition, Kaplan–Meier curves were used to detect whether the intersection genes showed a relationship with overall survival.

## 3. Results

### 3.1. Detection of Differentially Expressed miRNAs and Differentially Expressed mRNAs

**Figure 1:**
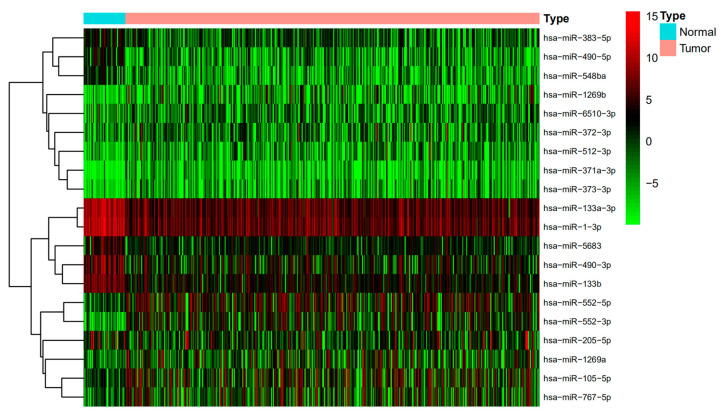
Clustering heatmap of differentially expressed miRNA.

**Figure 2:**
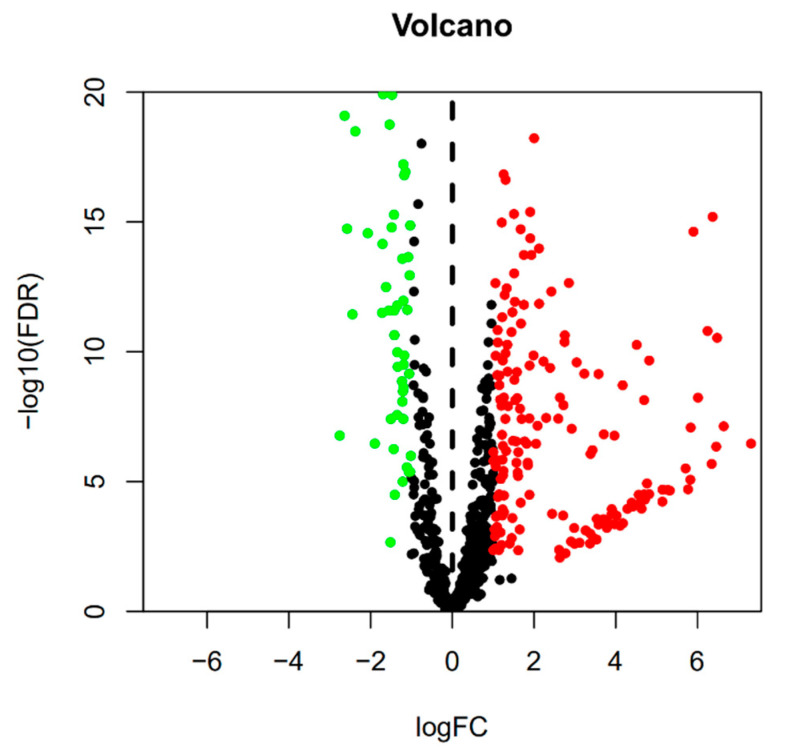
Volcanic maps of differentially expressed miRNAs.

The miRNA expression profiles displayed 267 differentially expressed miRNAs (DEmiRNAs) (185 up-regulated and 82 down-regulated) (Figure 1 and Figure 2). The mRNA expression profiles displayed 7531 differentially expressed mRNAs (DEmRNAs) (4395 up-regulated and 3136 down-regulated) (adjust *p*-value < 0.05 and |logFC| > 1.0).

### 3.2. Five miRNAs Associated with Overall Survival

**Figure 3:**
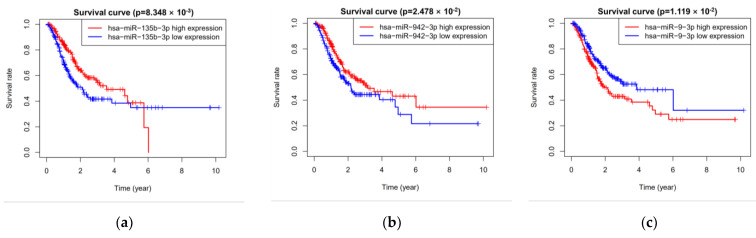

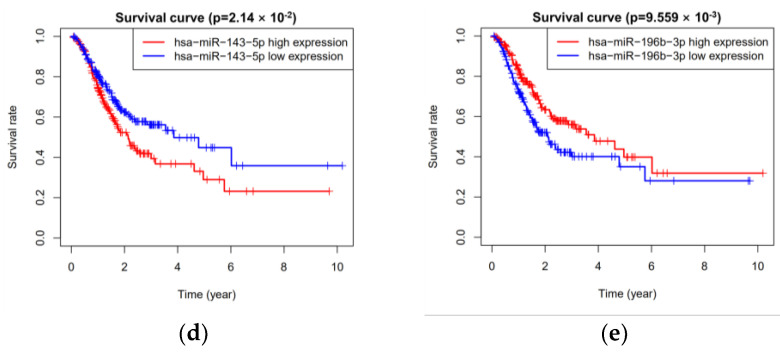
Five miRNAs associated with overall survival. (**a**) has-miR-135b-3p; (**b**) has-miR-942-3p; (**c**) has-miR-9-3p; (**d**) has-miR-143-5p; and (**e**) has-miR-196b-3p.

All 389 groups (miRNA expression profiles) were divided into training group (196) and testing group (193) randomly. Univariate Cox regression analysis revealed that fifteen miRNAs were related to overall survival in the training group. Multivariate Cox regression analysis selected five miRNAs (hsa-miR-9-3p, hsa-miR-135b-3p, hsa-miR-143-5p, hsa-miR-942-3p, and hsa-miR-196b-3p) from the fifteen miRNAs finally (Table 3). Besides, the Kaplan–Meier curve also showed that the five miRNAs were related to overall survival (Figure 3).

### 3.3. Prediction and Assessment of Five miRNAs for Overall Survival in Three Groups

**Figure 4:**
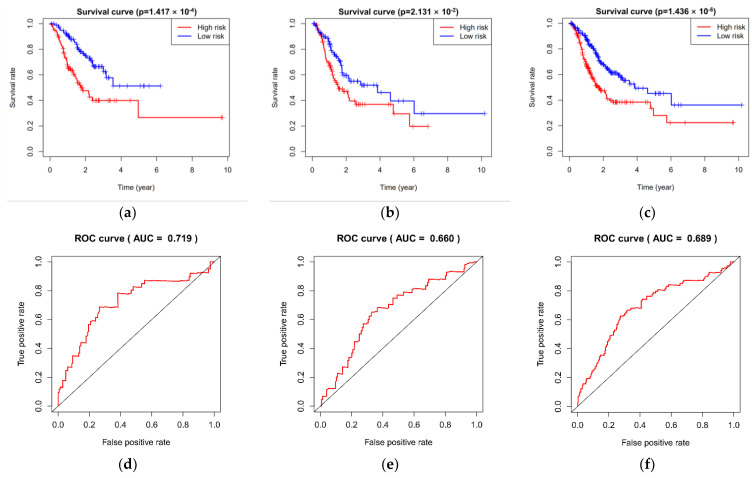

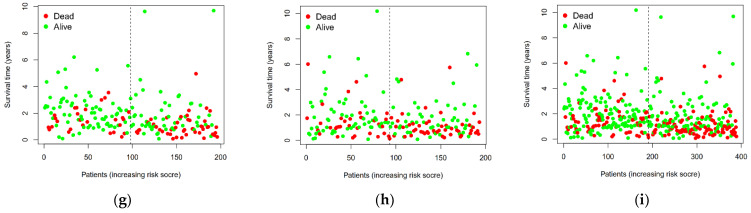
Verification and assessment of the five miRNAs. Kaplan–Meier curves in the (**a**) training group, (**b**) testing group, (**c**) whole group; The AUC curves in the (**d**) training group, (**e**) testing group, (**f**) whole group; Survival status of patients in high-risk and low-risk in the (**g**) training group, (**h**) testing group, (**i**) whole group.

According to the median value grouping of risk score, the Kaplan–-Meier curve displayed that the high-risk group had worse survival than the low-risk group in the training group (*p* = 1.417 × 10^−4^), the testing group (*p* = 2.131 × 10^−2^), and the whole group (*p* = 1.436 × 10^−5^; Figure 4a–c). The area under curve (AUC) of ROC for the five miRNAs severally attained 0.719, 0.660, and 0.689 in the training group, the testing group, and the whole group (Figure 4d–f), which indicated that the five miRNAs perform well in predicting the overall survival of gastric cancer patients. Furthermore, patients with high-risk scores had a higher death rate than those with low-risk scores in the three groups (Figure 4g–i).

### 3.4. Independence of the Five miRNAs

Based on the univariate and multivariate Cox regression analysis, the five miRNAs were related to the overall survival of patients (HR = 1.726, 95% CI = 1.396–2.136, *p* < 0.001). They were also independent in overall survival considering other clinical elements (HR = 1.971, 95% CI = 1.557–2.494, *p* < 0.001). Other clinical features include age, gender, stage, T stage, metastasis, and lymph node stage (Table 4).

### 3.5. Target Genes Prediction of Five miRNAs

**Figure 5:**
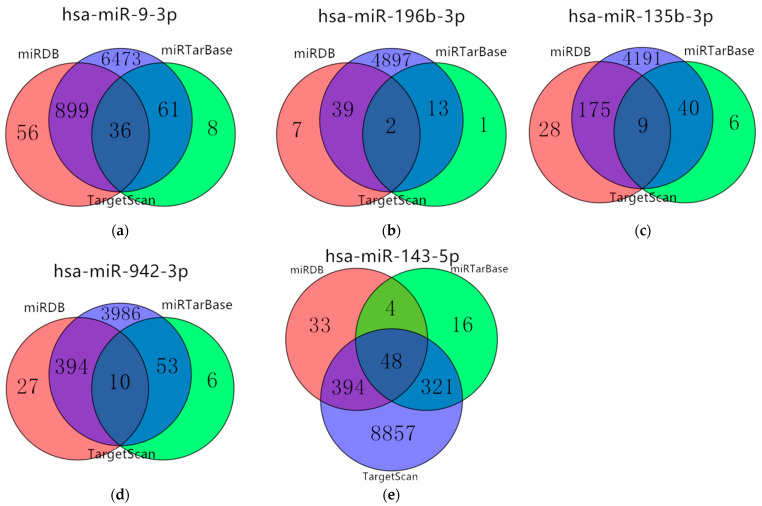
Venn diagram of target genes. (**a**) hsa-miR-9-3p; (**b**) hsa-miR-196-3p; (**c**) hsa-miR-135b-3p; (**d**) hsa-miR-942-3p; (**e**) hsa-miR-143-5.

The target genes were obtained and crosschecked in at least two databases. The predicted results showed that the five miRNAs (has-miR-9-3hashsa-miR-196hasp, hsa-miR-135b-3p, hsa-miR-942-3p, and hsa-miR-143-5p) overlapping target genes were 996, 54, 224, 457, and 767, respectively. The results were shown in Figure 5. Then, the above detected 7531 DEmRNAs (4395 up-regulated and 3136 down-regulated) were used to determine whether these target genes were involved in the development of gastric cancer.

**Figure 6:**
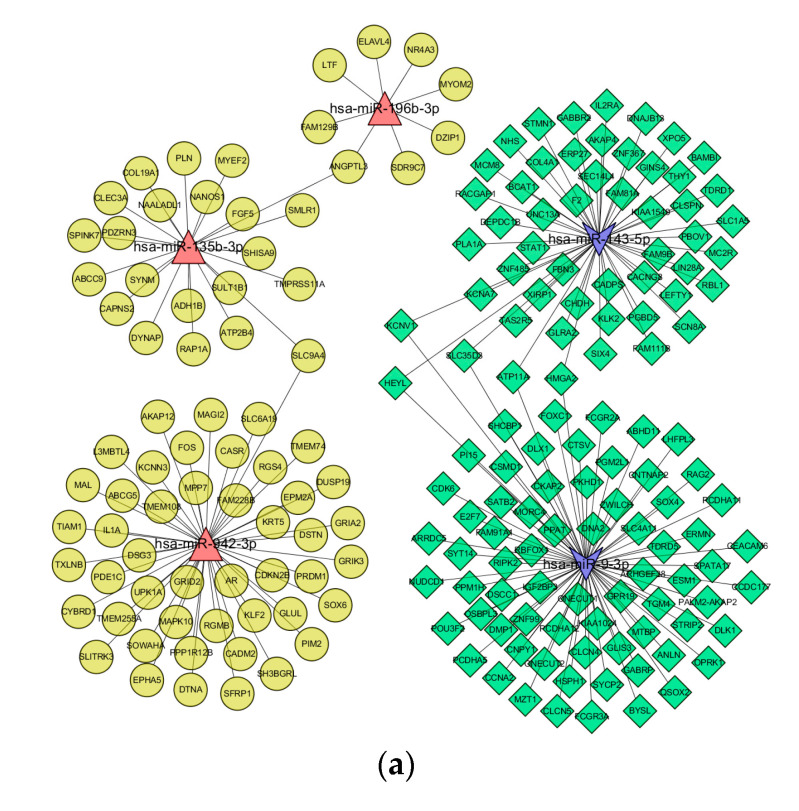

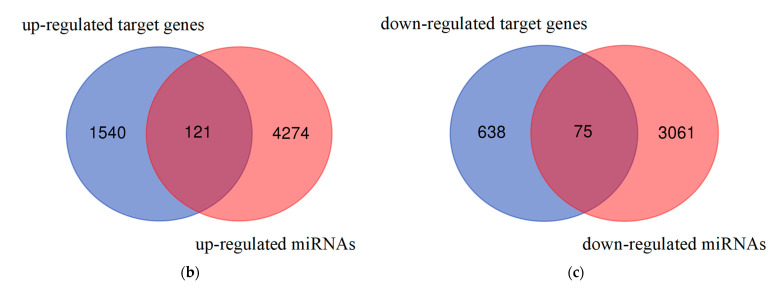
Network diagram of miRNAs regulating mRNAs. (**a**) Regulatory network between five miRNAs and 196 target genes. Red triangles mean 3 up-regulated miRNAs, blue arrows mean 2 down-regulated miRNAs, (**b**) green rhomboids mean 121 up-regulated mRNAs, and (**c**) yellow circles mean 75 down-regulated mRNAs.

Figure 6a displayed the regulatory network between five miRNAs and 196 target genes. There are 121 overlapping genes between the target genes of down-regulated miRNAs (hsa-miR-143-5p, hsa-miR-9-3p) (1661) and up-regulated mRNAs (4395). There were 75 overlapping genes between the target genes of up-regulated miRNAs (hsa-miR-135-3p, hsa-miR-196b-3p, hsa-miR-942-3p) (713) and down-regulated mRNAs (3136), as shown in Figure 6b,c.

### 3.6. Target Genes Functional Enrichment Analysis

**Figure 7:**
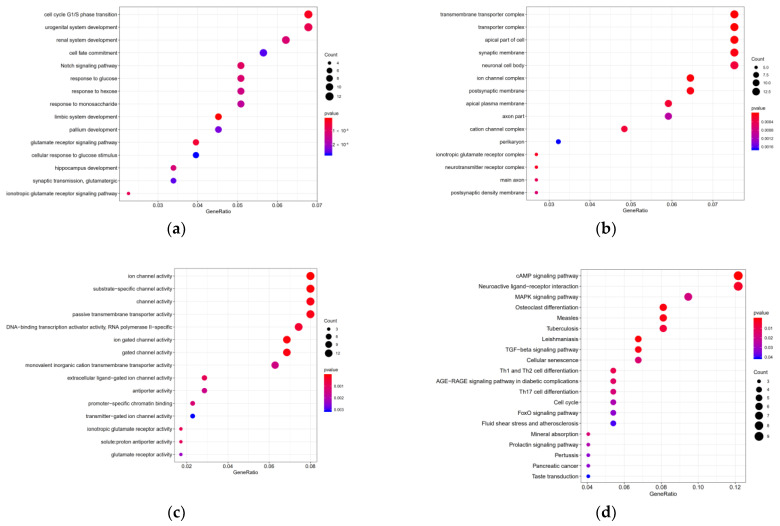
Functional enrichment analysis of target genes. (**a**) BP; (**b**) CC; (**c**) MF; (**d**) KEGG signaling pathways.

The GO results in the top fifteen terms, including biological process (BP), cellular component (CC), and molecular function (MF) were displayed in dot plot (Figure 7a–c). BP mainly contained cell cycle G1/S phase transition, urogenital and renal system development; CC mainly contained transmembrane transporter complex, transporter complex, and apical part of cell; MF mainly contained ion channel and substrate-specific channel activity. KEGG pathways analysis results were mainly enriched in the neuroactive ligand–receptor interaction, cAMP signaling pathway, and the MAPK signaling pathway (Figure 7d and Table 5).

### 3.7. Hub Genes of PPI Network and Survival Analysis of Target Genes

**Figure 8:**
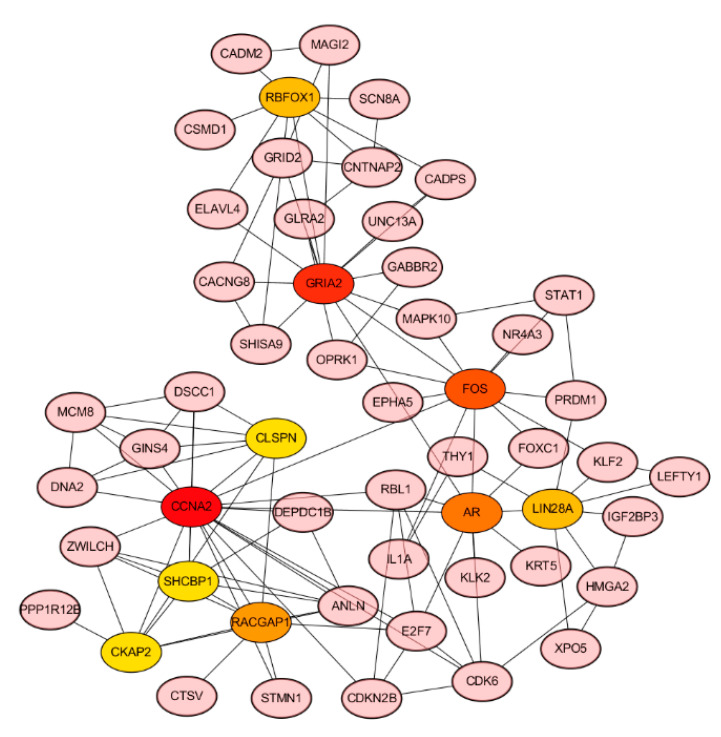
Core genes of PPI network.

**Figure 9:**
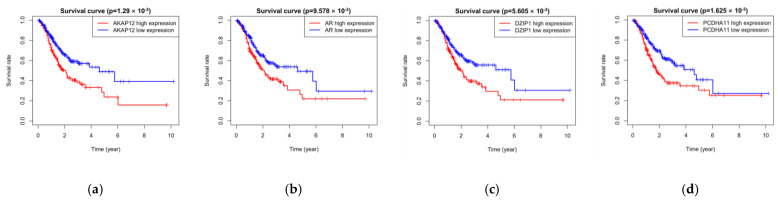

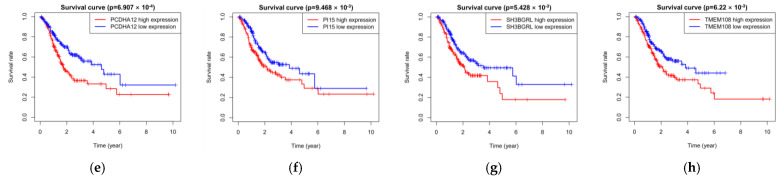
Eight target genes associated with overall survival. (**a**) AKAP12; (**b**) AR; (**c**) DEIP1/DZIP1; (**d**) PCDHA11; (**e**) PCDHA12; (**f**) P115; (**g**) SH3BGRL; (**h**) TMEM108.

The PPI network included a total of 196 target genes. The ten hub genes (CCNA2, GRIA2, FOS, AR, RACGAP1, RBFOX1, LIN28A, DSCC1, GRID2, OPRK1) from PPI network were screened by Cytoscape plug-in CytoHubba (Figure 8 and Table 6). Besides, the Kaplan–Meier curve indicated that the expression of eight genes (AKAP12, AR, DEIP1, PCDHA11, PCDHA12, P115, SH3BGRL, TMEM108) was correlated with survival prognosis (Figure 9).

### 3.8. The Working Mechanism of AR and Its Potential Relationship with the MAPK/ERK Signaling Pathway

**Figure 10:**
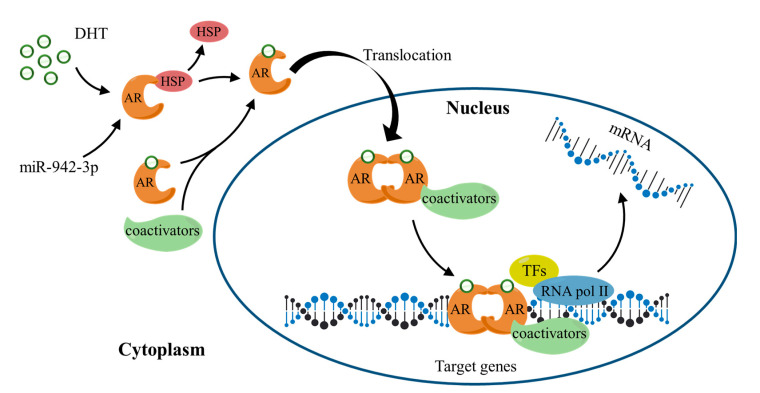
AR working mechanism. DHT: 5α-dihydrotestosterone; HSPs: heat shock proteins; TFs: transcription factors; RNA pol II: RNA polymerase II.

From the above, we can conclude that the Androgen Receptor (AR) was not only a hub gene in the PPI network but also associated with excessive survival of patients. AR can regulate the transcription of genes and express new proteins, ultimately changing the function of cells. Figure 10 shows a typical AR working mechanism. AR usually forms a complex with heat shock proteins (HSPs) in the cytoplasm. The binding of AR to androgen (such as 5α-dihydrotestosterone, DHT) alters its conformation, and HSPs are subsequently released. Under the action of coactivators, androgen–AR complexes are transferred to the nucleus and recognize androgen response elements in the form of homodimer to regulate downstream target gene expression.

**Figure 11:**
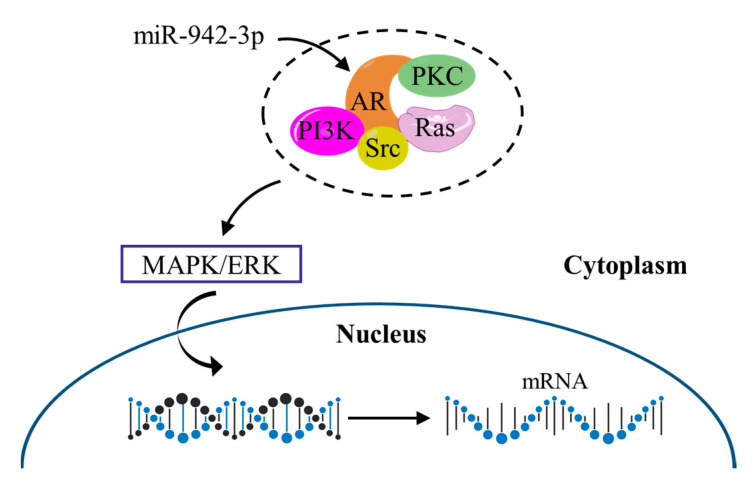
Potential relationship between the AR and MAPK/ERK signaling pathways. PI3K: phosphoinositide 3-kinase; Src: Src family kinase; RAS: Ras GTPase; PKC: protein kinase C.

In the absence of androgen, AR may depend on the MAPK/ERK signaling pathway to play its role. Figure 11 shows the potential relationship between the AR and MAPK/ERK signaling pathways. In the cytoplasm, AR can interact with several signaling molecules, including phosphoinositide 3-kinase (PI3K), Src family kinase (Src), Ras GTPase (Ras), and protein kinase C (PKC), which in turn converge on the MAPK/ERK pathway. Then, the MAPK/ERK enters the nucleus, where it translocates and interacts with transcription factors that regulate the expression of genes associated with cell proliferation.

## 4. Discussion

Gastric cancer is one of the most common tumors with high morbidity and mortality. Therefore, the detection of sensitive specific biomarkers for gastric cancer is urgent. Many studies indicated miRNAs could regulate expression in vivo, and it plays an essential role in the biological process of human malignancy [16]. Currently, some miRNAs have been used as potential prognostic indicators for tumors, such as miR-191 [17], miR-1908 [18], miR-217 [19], and miR-200c [20]. Previously, a variety of miRNAs were discovered in many prognostic markers for tumors [21,22], especially for gastric cancer [23].

In this study, we obtained 267 DEmiRNAs. All samples were divided into training group and testing group randomly. Then, the five miRNAs were constructed in the training group. At the same time, based on the median grouping of risk score, these five miRNAs were proved in the testing group and the whole group, respectively. Kaplan–Meier curves showed that overall survival was significantly lower in the high-risk group than in the low-risk group among the three groups. By ROC curve, the overall survival of the five miRNAs among the three groups showed better predictive ability. Subsequently, the Cox regression analysis indicated that the five miRNAs were independent of overall survival.

The target genes of five miRNAs were predicted in order to in-depth understand the regulatory mechanisms of these five miRNAs. GO analysis showed that the target genes were correlated with cell cycle G1/S phase transition, urogenital and renal system development, transmembrane transporter complex, transporter complex and apical part of the cell, ion channel, substrate-specific channel, and channel activity. The signaling pathways were enriched in the cAMP and MAPK signaling pathways and the Neuroactive ligand–receptor interaction. Park et al. pointed out that the cAMP signaling pathway inhibited the degradation of the HDAC8 and the expression of TIPRL in lung cancer cells, and also increased cisplatin-induced apoptosis [24]. Jagriti Pal et al. showed that the neuroactive ligand–receptor interaction pathway had a poor prognosis in patients with glioma [25]. The MAPK/ERK signaling pathway was essential in regulating cellular processes, such as cell differentiation, division, proliferation, and apoptosis.

The top ten hub genes (CCNA2, GRIA2, FOS, AR, RACGAP1, RBFOX1, LIN28A, DSCC1, GRID2, OPRK1) of target genes were detected by Cytoscape. Moreover, the Kaplan–Meier curve showed that eight target genes (AKAP12, AR, DEIP1, PCDHA11, PCDHA12, P115, SH3BGRL, TMEM108) were related to survival prognosis. Unexpectedly, AR was a hub gene in the PPI network, and it had a relationship with the excessive survival of patients. AR is a nuclear transcription factor, it can recognize and combine specific DNA sequences on target factors, thereby regulating the transcription of the gene and expressing new proteins, which ultimately changes the function of cells and promotes cell differentiation and the development of tissues and organs [26,27,28]. Salma S et al. showed that the p14ARF tumor suppressor could restrain AR activity and prevent apoptosis in prostate cancer cells [29]. Peng L et al. verified that AR could be directly combined with LAMA4, and it was related to enhanced cisplatin resistance in gastric cancer, providing a new mechanism for the treatment of drug-resistant gastric cancer [30]. In addition, AR may depend on the MAPK/ERK signaling pathway to function. Specifically, AR can interact with a variety of signaling molecules (PI3K, Src, Ras, and PKC) in the cytoplasm, which in turn converge on the MAPK/ERK pathway [31,32]. MAPK/ERK then enters the nucleus, where it translocates and interacts with transcription factors to regulate the expression of genes involved in cell proliferation [33].

In a word, this study found that the MAPK/ERK signaling pathway may help AR signal transduction and promote the interaction between AR and transcription factors, leading to cell proliferation. At the same time, AR is a target gene of the has-miR-942-3p, which well verifies the important role of the has-miR-942-3p in the occurrence and prognosis of gastric cancer.

## 5. Conclusions

This study built the five miRNAs (has-miR-9-3p, has-miR-135b-3p, has-miR-143-5p, has-miR-942-3p, has-miR-196-3p) prognostic modules, also verified and evaluated the prediction ability of the five miRNAs by grouping. They can be used as an independent factor in the prognosis of gastric cancer. By predicting the target genes to explore the potential biological functions, our results could provide a deeper understanding of the occurrence and development. This study identified the AR gene regulated by has-miR-942-3p which may depend on the MAPK/ERK signaling pathway to promote the proliferation of cancer cells. In future experiments, we will further explore the regulatory mechanisms of other miRNAs (has-miR-9-3p, has-miR-135b-3p, has-miR-143-5p, has-miR-196-3p) to provide effective prediction and treatment targets for gastric cancer patients.

## Figures and Tables

**Table 1:** The miRNA and mRNA expression profiles information.

	Variables	miRNA Expression Profiles	mRNA Expression Profiles
Case	Count	436	380
	Primary Site	Stomach	stomach
	Program	TCGA	TCGA
	Project	TCGA-STAD	TCGA-STAD
Files	Count	491	407
	Data Category	Transcriptome Profiling	Transcriptome Profiling
	Data Type	Isoform Expression Quantification	Gene Expression Quantification
	Workflow Type	BCGSC miRNA Profiling	HTSeq-Counts

**Table 2:** All patient information.

Variables		Case	Percentage (%)
Gender	Male	285	64.3
	Female	158	35.7
Age (years)	Range	30–90	
	Median	68	3.1
Futime (day)	Range	0–3720	
	Median	422	
Fustat	1	171	38.6
	0	272	61.3
Clinical stage	I	59	13.2
	II	130	29.2
	III	183	41.1
	IV	44	9.9
	Unknown	27	6
T stage	T1	23	5
	T2	93	20.8
	T3	198	44.6
	T4	119	26.7
	TX	10	2.2
Lymph node stage	N0	132	29.7
	N1	119	26.8
	N2	85	19.1
	N3	88	19.7
	NX	17	3.8
	Unknown	2	0.4
Metastatic	M0	391	88.2
	M1	30	6.7
	MX	22	4.9

**Table 3:** Univariate Cox regression and multivariate Cox regression of differentially expressed miRNAs.

ID	Univariate Cox Regression				Multivariate Cox Regression				
	HR	HR.95L	HR.95H	*p*-Value	Coef	HR	HR.95L	HR.95H	*p*-Value
hsa-miR-96-5p	0.761	0.642	0.903	0.002					
hsa-miR-7-5p	0.801	0.695	0.923	0.002					
hsa-let-7e-3p	1.379	1.112	1.71	0.003					
hsa-miR-143-5p	1.265	1.077	1.487	0.004	0.134	1.144	0.961	1.361	0.129
hsa-miR-942-3p	0.727	0.586	0.902	0.004	−0.178	0.837	0.663	1.056	0.132
hsa-miR-183-5p	0.806	0.69	0.942	0.007					
hsa-miR-196b-3p	0.648	0.468	0.897	0.009	−0.307	0.736	0.527	1.027	0.072
hsa-miR-125a-5p	1.401	1.067	1.839	0.015					
hsa-miR-135b-3p	0.799	0.665	0.96	0.017	−0.148	0.862	0.706	1.052	0.144
hsa-miR-30a-3p	1.21	1.024	1.428	0.025					
hsa-miR-652-5p	0.784	0.623	0.986	0.037					
hsa-miR-9-3p	1.17	1.008	1.359	0.039	0.147	1.159	0.989	1.358	0.069
hsa-miR-99a-3p	1.175	1.007	1.372	0.040					
hsa-miR-139-5p	1.221	1.007	1.48	0.042					
hsa-miR-137-3p	1.16	1.000	1.346	0.049					

**Table 4:** Univariate and multivariate Cox regression of clinical features.

Clinical Features	Univariate Cox Regression				Multivariate Cox Regression			
	HR	HR.95L	HR.95H	*p*-Value	HR	HR.95L	HE.95H	*p*-Value
Age	1.015	0.999	1.032	0.062	1.027	1.010	1.045	0.002
Gender	1.225	0.853	1.760	0.271	1.510	1.027	2.218	0.036
Grade	1.278	0.908	1.800	0.160	1.115	0.781	1.591	0.550
Stage	1.607	1.294	1.996	<0.001	1.210	0.807	1.815	0.357
T	1.288	1.038	1.599	0.022	1.215	0.911	1.621	0.186
M	1.880	1.013	3.489	0.045	1.818	0.844	3.917	0.127
N	1.361	1.170	1.584	<0.001	1.233	0.987	1.540	0.065
riskScore	1.726	1.395	2.136	<0.001	1.971	1.557	2.494	<0.001

**Table 5:** KEGG signaling pathways of the target genes.

ID	Description	*p*-Value	*Q*-Value	Count	Gene
hsa04024	cAMP signaling pathway	0.0002	0.0272	9	TIAM1/FOS/GRIA2/MAPK10/PLN/MC2R/ATP2B4/GABBR2/RAP1A
hsa05140	Leishmaniasis	0.0007	0.0603	5	FCGR3A/FOS/STAT1/IL1A/FCGR2A
hsa04380	Osteoclast differentiation	0.0011	0.0603	6	FCGR3A/FOS/MAPK10/STAT1/IL1A/FCGR2A
hsa05162	Measles	0.0017	0.0603	6	CDK6/FOS/MAPK10/STAT1/IL1A/IL2RA
hsa04350	TGF-beta signaling pathway	0.0017	0.0603	5	CDKN2B/RGMB/LEFTY1/BAMBI/RBL1
hsa04080	Neuroactive ligand-receptor interaction	0.0039	0.1148	9	GRIA2/GRID2/MC2R/F2/GLRA2/GABRP/GRIK3/GABBR2/OPRK1
hsa05152	Tuberculosis	0.0062	0.1570	6	FCGR3A/MAPK10/RIPK2/STAT1/IL1A/FCGR2A
hsa04658	Th1 and Th2 cell differentiation	0.0101	0.2256	4	FOS/MAPK10/STAT1/IL2RA
hsa04933	AGE-RAGE signaling pathway	0.0135	0.2506	4	MAPK10/STAT1/IL1A/COL4A1
hsa04218	Cellular senescence	0.0159	0.2506	5	CDK6/CDKN2B/IL1A/CCNA2/RBL1
hsa04978	Mineral absorption	0.0162	0.2506	3	SLC6A19/CYBRD1/ATP2B4
hsa04659	Th17 cell differentiation	0.0169	0.2506	4	FOS/MAPK10/STAT1/IL2RA
hsa04010	MAPK/ERK signaling pathway	0.0187	0.2554	7	CACNG8/FOS/MAPK10/IL1A/STMN1/FGF5/RAP1A
hsa04917	Prolactin signaling pathway	0.0266	0.3244	3	FOS/MAPK10/STAT1
hsa04110	Cell cycle	0.0274	0.3244	4	CDK6/CDKN2B/CCNA2/RBL1
hsa04068	FoxO signaling pathway	0.0326	0.3247	4	CDKN2B/MAPK10/KLF2/RAG2
hsa05133	Pertussis	0.0329	0.3247	3	FOS/MAPK10/IL1A
hsa05212	Pancreatic cancer	0.0329	0.3247	3	CDK6/MAPK10/STAT1
hsa05418	Fluid shear stress and atherosclerosis	0.0392	0.3471	4	FOS/MAPK10/KLF2/IL1A
hsa04742	Taste transduction	0.0410	0.3471	3	PDE1C/TAS2R5/GABBR2

**Table 6:** Identification of the top ten core genes.

Node_Name	MCC	DMNC	MNC	Degree	EPC	Bottle Neck	Ec Centricity	Closeness	Radiality	Betweenness	Stress	Clustering Coefficient
CCNA2	236	0.264	18	18	35.308	19	0.097	43.961	7.799	2843.324	5374	0.235
GRIA2	22	0.233	8	13	33.492	71	0.129	44.383	8.065	3792.232	8494	0.128
FOS	17	0.220	7	12	34.894	84	0.111	46.076	8.159	3863.375	7988	0.091
AR	33	0.329	7	10	35.226	18	0.111	43.369	7.987	2490.437	5478	0.200
RACGAP1	131	0.408	8	9	33.683	2	0.086	33.073	7.110	210.833	484	0.389
RBFOX1	12	0.238	6	8	28.267	4	0.111	35.586	7.525	953.058	2332	0.179
LIN28A	9	0.309	3	8	28.652	3	0.097	36.095	7.517	790.409	2030	0.107
DSCC1	28	0.454	5	7	30.653	3	0.086	32.073	7.094	392.500	704	0.333
GRID2	13	0.285	6	7	28.277	2	0.111	34.919	7.525	366.790	918	0.286
OPRK1	10	0.463	3	7	25.901	13	0.097	37.251	7.721	1633.949	3034	0.143

## Data Availability

The miRNA expression profiles, mRNA expression profiles data, and clinical information of all gastric cancer samples were gained from The Cancer Genome Atlas (TCGA) database (https://www.cancer.gov (accessed on 20 August 2022)).
